# Comparison of the biological properties of bat-derived filovirus envelope glycoproteins

**DOI:** 10.1128/jvi.01018-25

**Published:** 2025-09-25

**Authors:** Francois Edidi-Atani, Yannick Munyeku Bazitama, Hiroko Miyamoto, Akina Mori-Kajihara, Hayato Sugiura, Manabu Igarashi, Jean Jacques Muyembe-Tamfum, Steve Ahuka-Mundeke, Ayato Takada

**Affiliations:** 1 Division of Global Epidemiology, International Institute for Zoonosis Control, Hokkaido University 12810, Sapporo, Japan; 2 Institut National de Recherche Biomédicale 309878, Kinshasa, Democratic Republic of Congo; 3 Département de Biologie Médicale, Faculté de Médecine, Université de Kinshasa 58820, Kinshasa, Democratic Republic of Congo; 4 Faculty of Medicine, University of Kikwit 601651, Kikwit, Democratic Republic of Congo; 5 International Collaboration Unit, International Institute for Zoonosis Control, Hokkaido University 12810, Sapporo, Japan; 6 One Health Research Center, Hokkaido University 12810, Sapporo, Japan; 7 Department of Disease Control, School of Veterinary Medicine, University of Zambia 108234, Lusaka, Zambia; The Ohio State University, Columbus, Ohio, USA

**Keywords:** Lloviu virus, Bombali virus, Mengla virus, Dehong virus, Ebola virus, Marburg virus, filovirus, glycoprotein, bat

## Abstract

Although some filoviruses, such as Ebola virus (EBOV) and Marburg virus (MARV), are highly pathogenic in humans, novel filoviruses, including Lloviu virus (LLOV), Bombali virus (BOMV), Mengla virus (MLAV), and Dehong virus (DEHV), whose biological properties are poorly understood, have been found in bats. In this study, we characterized the envelope glycoproteins (GPs) of these bat-derived filoviruses (BatFiloVs). We first confirmed that virus-like particles consisting of their GPs, nucleoproteins, and matrix proteins were filamentous. Interestingly, although BatFiloVs were serologically distinct, some previously established monoclonal antibodies (MAbs) (e.g., 6D6) successfully neutralized vesicular stomatitis Indiana viruses pseudotyped with LLOV, BOMV, or DEHV GPs. The pseudotyped viruses bearing BatFiloV GPs utilized human TIM-1 and C-type lectins for entry into cells, although the efficiency tended to be lower than for EBOV and/or MARV GP-pseudotyped viruses. These viruses broadly infected cultured cells derived from various animal species, including humans and bats. However, viruses pseudotyped with DEHV and MARV GPs failed to infect the Yaeyama flying fox cell line, whereas the other pseudotyped viruses infected this cell line. Interestingly, the virus bearing BOMV GP showed the greatest ability to infect cell lines derived from Angolan free-tailed bats, the only known host species of BOMV. We identified unique amino acid residues at the interface between GP and its receptor (i.e., Niemann-Pick C1), which might explain these differences. Our results suggest that the biological properties of filovirus GPs are generally consistent with their phylogenetic relationship and that BatFiloVs may have differential pathogenicity and host range restriction.

**IMPORTANCE:**

Filoviruses, such as EBOV and MARV, are known to cause severe hemorrhagic fever in humans and nonhuman primates. With the recent advancements in next-generation sequencing, novel filoviruses have been detected in bats. However, their pathogenicity and host tropism remain largely unknown. Here, we focus on the filovirus spike protein GP, which plays a crucial role in the viral lifecycle, and discuss the biological properties of BatFiloVs. We studied the primary structures of GPs, virus particle morphology, antigenic differences of GPs, neutralizing capacities of anti-EBOV and -MARV GP MAbs, usage of some attachment factors during the entry into cells, and GP-mediated cellular tropism. The present study provides fundamental information for understanding the BatFiloV ecology, host ranges, and potential risks as zoonotic pathogens for humans. This knowledge will guide public health interventions to prevent virus spillovers and the development of surveillance strategies and specific countermeasures.

## INTRODUCTION

Filoviruses belong to the family *Filoviridae*, which includes nine genera**:** *Orthoebolavirus, Orthomarburgvirus, Cuevavirus, Oblavirus, Striavirus, Thamnovirus, Tapjovirus*, *Dianlovirus,* and *Loebevirus,* comprising 17 species and 18 viruses (1, 2). Of these, Ebola virus (EBOV), Sudan virus (SUDV), Tai Forest virus (TAFV), and Bundibugyo virus (BDBV) in the genus *Orthoebolavirus*, and Marburg virus (MARV) and Ravn virus (RAVV) in the genus *Orthomarburgvirus,* are known to be pathogenic in humans and cause severe hemorrhagic fever with high mortality rates. Reston virus (RESTV), which causes lethal infection in nonhuman primates, is believed to be nonpathogenic to humans, although its pathogenicity has not yet been fully clarified (3). Almost all filovirus disease outbreaks have occurred in Equatorial Africa, except for some imported cases reported in Europe and the United States, suggesting that diseases caused by filoviruses pathogenic to humans are endemic primarily in the African continent (4). The natural reservoir hosts for filoviruses are not fully elucidated, except for MARV, which has been frequently isolated from or detected in Egyptian fruit bats (*Rousettus aegyptiacus*) (5–7).

The recent advancement of new pathogen detection tools, such as next-generation sequencing, has led to the expansion of the *Filoviridae* family with the discovery of new filoviruses in bats: Lloviu virus (LLOV) in the genus *Cuevavirus*, Bombali virus (BOMV) in the genus *Orthoebolavirus,* Mengla virus (MLAV), and Dehong virus (DEHV) in the genus *Dianlovirus* (1, 2). Among these bat-derived filoviruses (BatFiloVs), the Lloviu virus (LLOV) genome was first detected in insectivorous bats (*Miniopterus schreibersii*) in some European countries (8–10), and infectious LLOV was subsequently isolated in the same bat species in Hungary and Italy (11, 12). The Bombali virus (BOMV) genome was detected in free-tailed bats (*Mops condylurus* and *Chaerephon pumilus*) in several African countries (13–16). The Mengla virus (MLAV) genome was detected in fruit bats (*Rousettus leschenaultii*) in China (17). More recently, Dehong virus (DEHV) was isolated from fruit bats (*Rousettus leschenaultii*) in China (18).

Known filovirus particles are enveloped, variously shaped, but predominantly filamentous and contain a linear, negative-sense, and non-segmented RNA genome. The RNA genomes of the above-mentioned filoviruses encode at least seven structural proteins: envelope glycoprotein (GP), major matrix protein (VP40), nucleoprotein (NP), polymerase cofactor (VP35), replication/transcription protein (VP30), minor matrix protein (VP24), and RNA-dependent RNA polymerase (L) (2). In addition to these structural proteins, orthoebolaviruses express a nonstructural soluble GP (sGP and ssGP) through RNA editing (2, 19–21). Among these viral proteins, GP is the sole protein present on the virus surface and is responsible for receptor binding and fusion of the virus envelope with the host cell membrane (22, 23). This protein is the only target of neutralizing antibodies to filoviruses (24). It exhibits genetic and antigenic variation, whereas other filovirus proteins (e.g., NP and VP40) are relatively conserved among filovirus species (25). Thus, GP is expected to induce more species-specific and less cross-reactive antibodies than other filovirus proteins (26). The GP monomer consists of 2 subunits, GP1 and GP2, which are linked by a disulfide bridge (27). The GP1 subunit contains a receptor-binding domain (RBD) responsible for attachment to the host cell receptor Niemann-Pick C1 (NPC1) and the mucin-like domain (MLD), which is heavily glycosylated with large amounts of N- and O-linked glycans (28–30). Its highly variable amino acid sequences and sugar chain structures suggest different GP properties among filovirus species (23). During viral entry into cells, GP1 is digested with host proteases to expose RBD, followed by the interaction with NPC1 on the endosomal membrane (31). The GP2 subunit contains an internal fusion loop, two heptad repeats, a membrane-proximal external region, a transmembrane domain, and a cytoplasmic tail (32).

Currently, BatFiloVs are poorly characterized since infectious viruses have rarely been isolated. Their biological properties, such as pathogenicity in humans and nonhuman primates, transmission routes, and host range remain largely unknown, whereas it was reported that immunodeficient mice infected with recombinant LLOV or BOMV showed low pathogenicity or minimal signs of diseases (15, 33) and that ferrets survived without signs of disease regardless of the dose and exposure routes after LLOV infection (34). In the present study, we focused on filovirus envelope GP, which is thought to play a crucial role in viral pathogenicity and tropism, and compared biological properties among mammalian filoviruses (i.e., BatFiloVs and human/nonhuman primate-pathogenic filoviruses). We first analyzed the primary structures of the GPs, the morphology of virus-like particles (VLPs) consisting of GP, NP, and VP40, and the antigenic differences of GPs among filoviruses. Then, using replication-incompetent vesicular stomatitis Indiana virus (VSIV) pseudotyped with filovirus GPs, we investigated the neutralizing activities of several previously established monoclonal antibodies (MAbs) against EBOV and MARV GPs, the ability of GPs to use host attachment factors, and cellular tropism in cell lines derived from various animal species. Our findings provide valuable insights into the functional properties of BatFiloV GPs and contribute to a better understanding of their zoonotic risk.

## RESULTS

### Comparison of the primary structures among BatFiloV, EBOV, and MARV GPs

**Fig 1:**
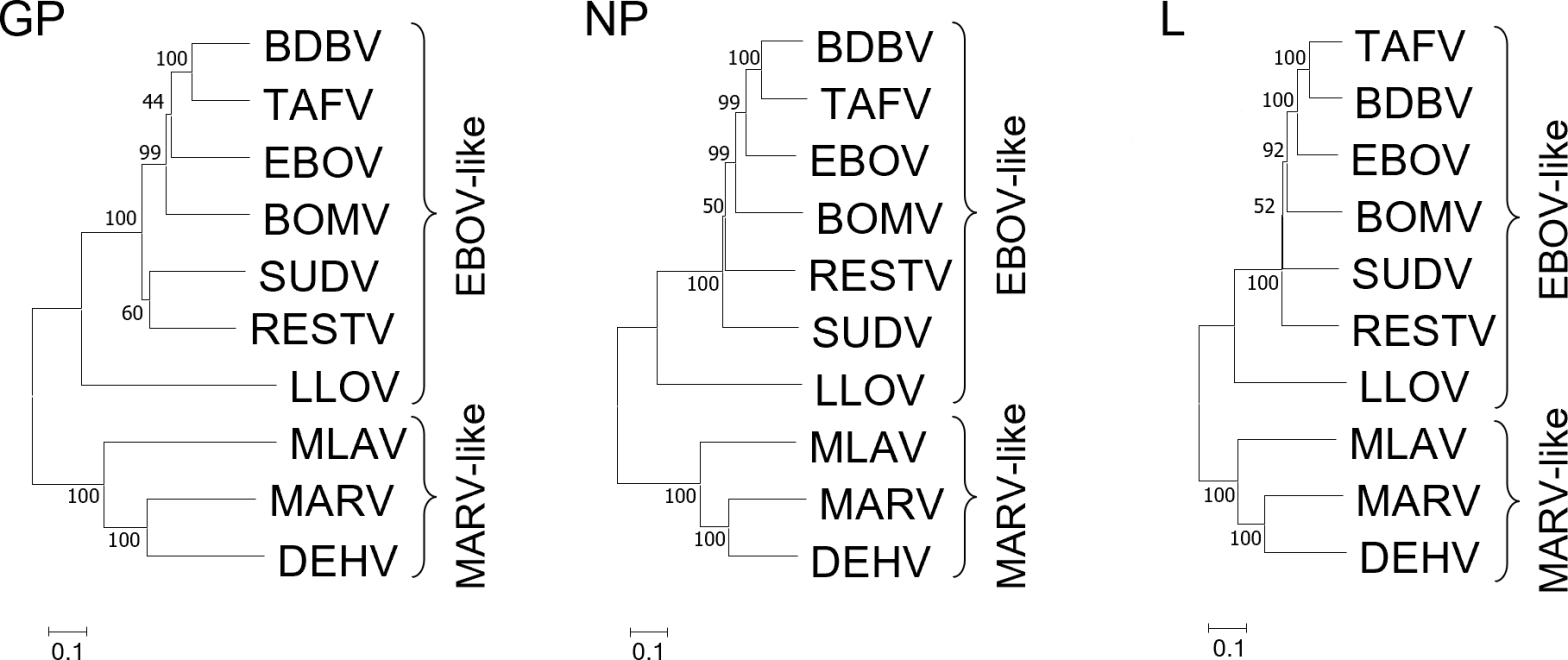
Phylogenetic analysis of mammalian filoviruses based on GP, NP, and L amino acid sequences. Phylogenetic trees were constructed using the neighbor-joining method as described in Materials and Methods (sequence analyses).

In the phylogenetic tree of mammalian filoviruses, GPs are divided into EBOV-like and MARV-like phylogroups, like other proteins such as NP and L, as described previously (18) (Fig. 1). We first compared the primary structures of BatFiloV, EBOV, and MARV GPs (Fig. S1 and S2). As expected, the N-terminal one-third regions and C-terminal one-third regions were relatively conserved among the viruses, and the middle regions, principally corresponding to their MLDs, were highly divergent. We identified 10 highly conserved cysteine residues in the relatively conserved regions. Additionally, two cysteine residues in GP1 (C121 and C147) were conserved only among EBOV, LLOV, and BOMV. We found that cysteine residues contributing to the disulfide bridge that links GP1 and GP2, as well as those involved in intramolecular subunit stabilization, were conserved (35). Two cysteine residues (C670 and C672), known to be required for GP acylation, were also conserved (36). The potential cleavage site motifs recognized by ubiquitous host proteases such as furin were found in all the GP sequences: RRRR for LLOV as previously described (37), RAKR for BOMV, RKRR for DEHV, and two motifs (RSKR and KKKR) for MLAV. N-glycosylation motifs were also found in all GPs: 17 sites for EBOV and 9 sites for BOMV as described in a previous study (38), 20 sites for LLOV, 23 sites for MARV, 15 sites for MLAV, and 19 sites for DEHV. We found two fully conserved N-glycosylation sites in GP2 (N563 and N618) among all orthoebolaviruses, MARV, and LLOV (38). The O-glycosylation site prediction revealed 80 sites for EBOV, 88 sites for LLOV, 61 sites for BOMV, 100 sites for MARV, 59 sites for MLAV, and 97 sites for DEHV, most of which were likely involved in forming their MLDs (Fig. S1). LLOV, MARV, MLAV, and DEHV MLDs were located over the cleavage sites. Consistent with the phylogenetic relationships, the overall characteristics of GP primary structures of LLOV and BOMV were closer to EBOV than to MARV, whereas MLAV and DEHV were closer to MARV than to EBOV.

### Morphology of VLPs consisting of BatFiloV GP, VP40, and NP

**Fig 2:**
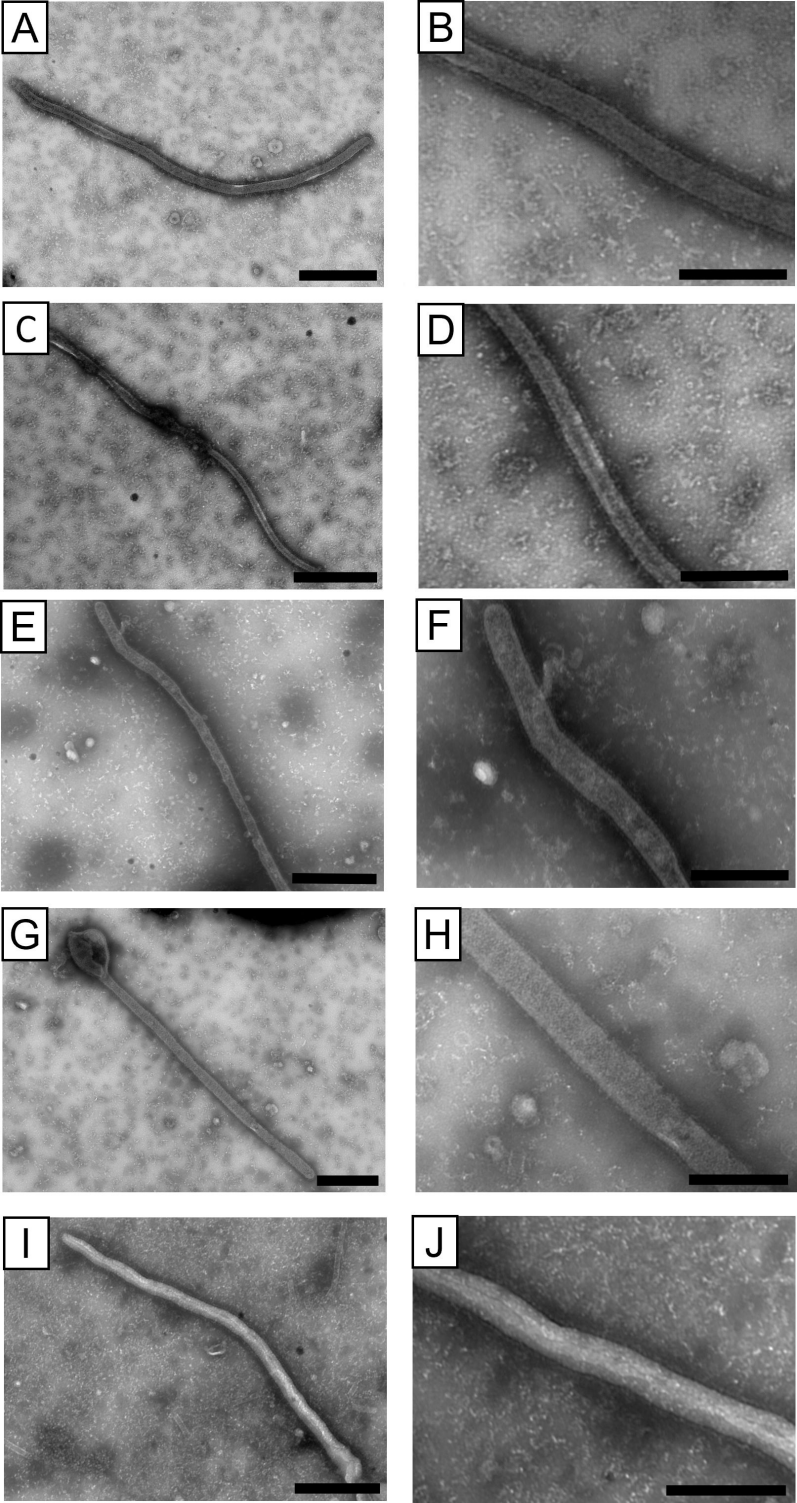
Transmission electron microscopy of VLPs. VLPs purified from the supernatant of HEK293T cells transfected with plasmids encoding GP, NP, and VP40 of LLOV (**A and B**), BOMV (**C and D**), MLAV (**E and F**), DEHV (**G and H**), and EBOV (**I and J**) were stained as described in Materials and Methods. Scale bars represent 500 nm (A, C, E, G, and I) and 200 nm (B, D, F, H, and J).

A unique characteristic of filovirus particles is their filamentous shape, and it has been shown that morphologically similar VLPs can be produced by the expression of GP, VP40, and NP (37, 39). However, information on the morphology of BatFiloV particles remains limited. To address this, we generated VLPs by transient expression of BatFiloV GP, VP40, and NP in cultured cells and examined their morphology using electron microscopy (Fig. 2; Fig. S3). We confirmed that VLPs composed of the LLOV, BOMV, MLAV, or DEHV proteins exhibited a filamentous shape with densely arrayed spikes on their surfaces, similar to those of EBOV. Their diameters were uniform (approximately 80–90 nm), whereas their length varied. BOMV VLPs tended to be slightly thinner. These results suggest that BatFiloV GP, VP40, and NP also play crucial roles in producing virus particles with the characteristic filamentous shape.

### Antigenic comparison among filovirus GPs

**Fig 3:**
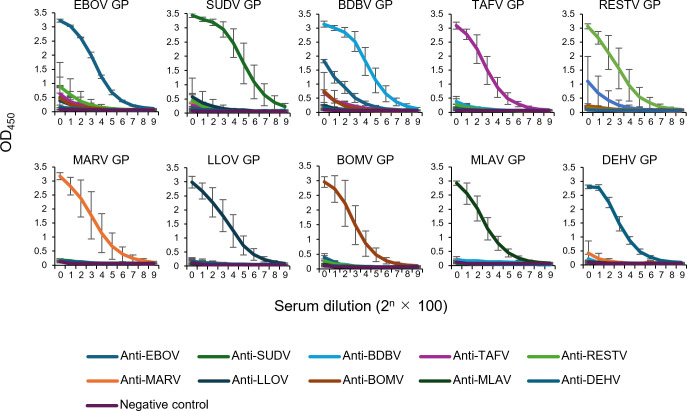
Cross-reactivities of anti-GP sera among filoviruses in ELISA. Two-fold serial dilutions of mouse antisera to VLPs comprising EBOV, SUDV, BDBV, TAFV, RESTV, MARV, LLOV, BOMV, MLAV, and DEHV proteins were tested for IgG reactivity to purified GP antigens of the respective viruses. Three mice were used for each virus, and the averages and standard deviations are shown.

Mammalian filoviruses are phylogenetically divided into four genera: *Orthoebolavirus*, *Orthomarburgvirus*, *Cuevavirus*, and *Dianlovirus* (2), three of which (*Orthoebolavirus*, *Cuevavirus*, and *Dianlovirus*) include BatFiloVs. However, information on the antigenic differences among BatFiloV GPs and other filovirus GPs remains limited (40). To investigate antigenic relationships among GPs of mammalian filoviruses, we produced mouse antisera against VLPs of ten mammalian filoviruses selected from each genus and tested their IgG reactivities to the respective GP antigens (Fig. 3). We found that anti-LLOV, anti-BOMV, anti-MLAV, and anti-DEHV GP sera showed exclusive reactivity to their homologous GP antigens. Anti-EBOV sera showed slight cross-reactivity with BDBV and RESTV GPs. In general, all antisera demonstrated high specificity to homologous GP antigens with limited cross-reactivity to other viral antigens.

### Cross-reactivity of anti-EBOV and anti-MARV GP MAbs against BatFiloVs

**Fig 4:**
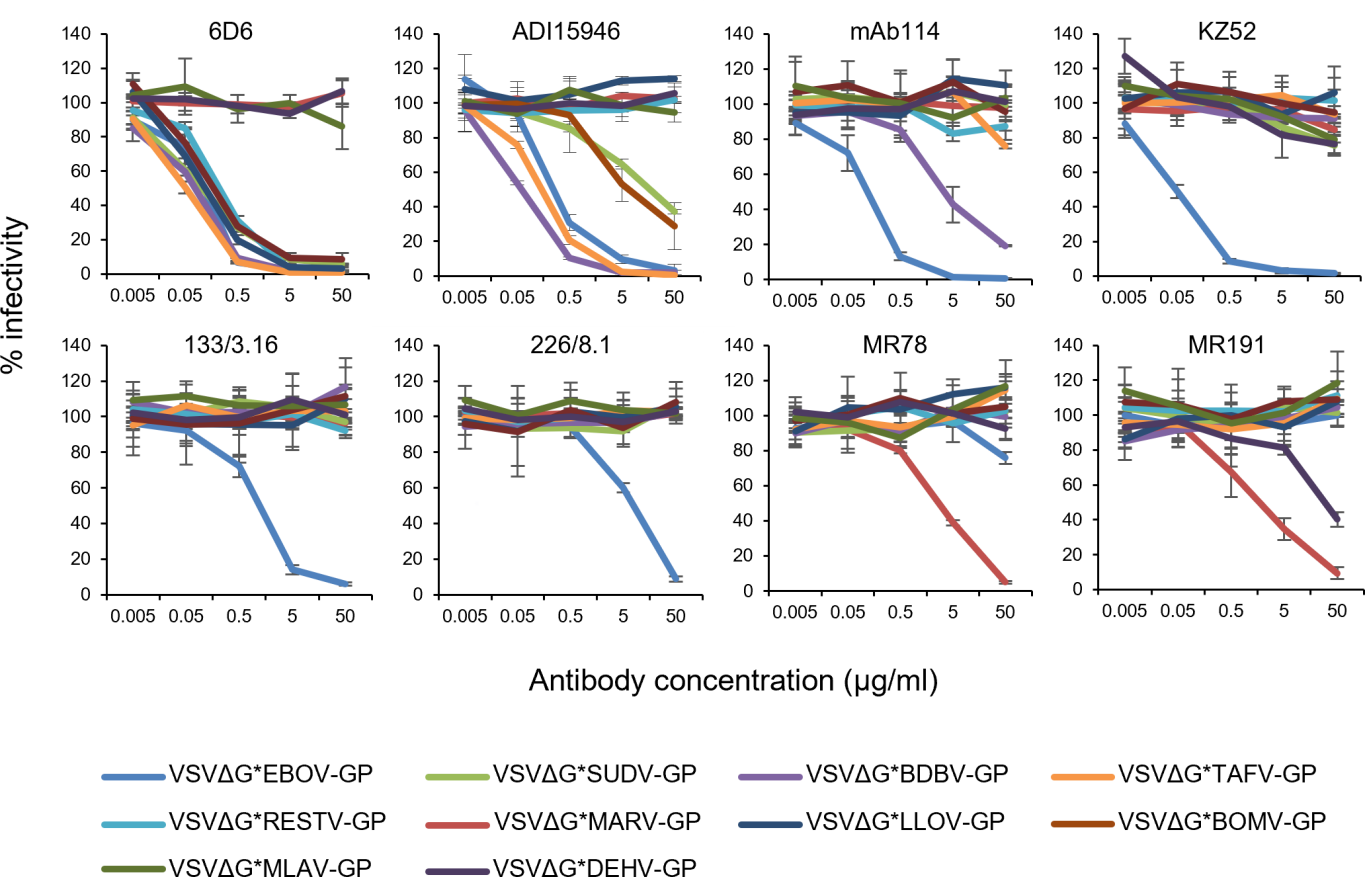
Cross-neutralizing potential of MAbs against BatFiloVs. Vero E6 cells were infected with pseudotyped viruses mixed with serial dilutions (0.005–50 µg/mL) of 6D6, ADI15946, mAb114, KZ52, 133/3.16, 226/8.1, MR78, and MR191 as described in Materials and Methods. Averages and standard deviations of three independent experiments are shown.

The recent discovery of novel filoviruses highlights the urgent need to develop compounds or drugs for pan-filovirus treatment. Therapeutic use of neutralizing MAbs is a potential option in the event of BatFiloV emergence in the human population (41). In this study, some previously established MAbs targeting EBOV and MARV GPs were tested for their ability to neutralize other filoviruses, including BatFiloVs, using VSIV pseudotyped (VSVΔG*) with filovirus GPs (Fig. 4). We found that 6D6, which has been shown to inhibit EBOV, SUDV, BDBV, TAFV, and RESTV infection (42), efficiently neutralized VSVΔG*LLOV-GP and VSVΔG*BOMV-GP but not VSVΔG*MLAV-GP, VSVΔG*DEHV-GP, or VSVΔG*MARV-GP. ADI-15946, which is known to inhibit EBOV, BDBV, and TAFV infection (43, 44), efficiently neutralized VSVΔG*BDBV-GP and VSVΔG*TAFV-GP and showed slight neutralization of VSVΔG*BOMV-GP and VSVΔG*SUDV-GP but did not neutralize the other viruses. mAb114, a drug approved for EBOV treatment (45), showed strong inhibitory activity against VSVΔG*EBOV-GP and slightly neutralized VSVΔG*BDBV-GP. Previously reported EBOV-specific KZ52, 133/3.16, and 226/8.1 (24, 46) showed no cross-neutralizing activity against the viruses with other filovirus GPs. Of the two anti-MARV GP neutralizing MAbs, MR78 and MR191 (47–49), MR191 neutralized VSVΔG*DEHV-GP to some extent, but not VSVΔG*MLAV-GP. These findings indicate that the epitope of 6D6 is widely shared among LLOV, BOMV, and other orthoebolaviruses and that MR191 recognizes a common epitope partially conserved between DEHV and MARV GPs.

### Roles of cellular attachment factors in BatFiloV entry

**Fig 5:**
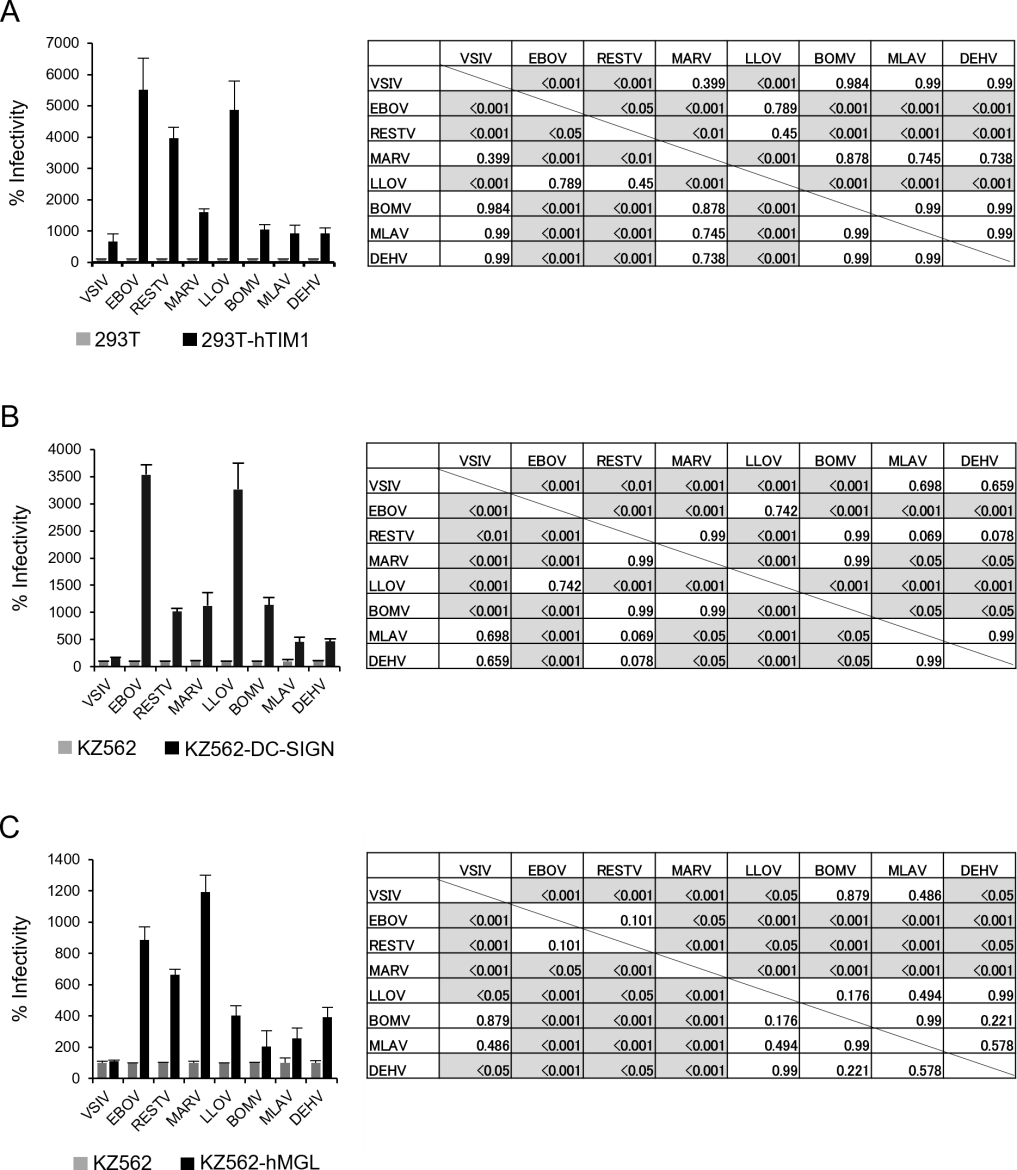
Infectivities of pseudotyped viruses in cells expressing viral attachment factors. VSVΔG*VSIV-G, VSVΔG*EBOV-GP, VSVΔG*RESTV-GP, VSVΔG*MARV-GP, VSVΔG*LLOV-GP, VSVΔG*BOMV-GP, VSVΔG*MLAV-GP, and VSVΔG*DEHV-GP were inoculated into control HEK293T cells and HEK293T cells expressing hTIM-1 (**A**). These viruses were also inoculated into control K562 cells and K562 cells expressing DC-SIGN (**B**) or hMGL (**C**). Relative percentages of infectivity were calculated by setting IUs in infected control cells to 100%. Each experiment was performed three times, and averages and standard deviations are shown. One-way analysis of variance (ANOVA) was used to compare infectivity among viruses. If the overall ANOVA indicated a significant group effect (*P* < 0.05), a post hoc Tukey’s honestly significant difference (Tukey HSD) test was performed to evaluate pairwise group comparisons while adjusting for multiple testing. The statistical analyses and data manipulations were carried out using the tidyverse and stats packages in R (version 4.4.2). *P* values for each comparison are shown in the tables, and shadowed cells represent statistically significant differences.

Human T-cell immunoglobulin and mucin domain-1 (hTIM-1) has been shown to facilitate the attachment of EBOV and MARV to cell surfaces (50). We investigated the potential of hTIM-1 to promote BatFiloV entry into cells using pseudotyped viruses (Fig. 5A). All tested viruses were found to infect hTIM-1-expressing cells more efficiently than control cells, although the extent of enhancement varied among the viruses. VSVΔG*LLOV-GP, like VSVΔG*EBOV-GP, showed an approximately 50-fold increase in infectivity in hTIM-1-expressing cells compared with control cells. On the other hand, the infectivities of VSVΔG*BOMV-GP, VSVΔG*MLAV-GP, VSVΔG*DEHV-GP, and VSVΔG*MARV-GP were not enhanced as significantly as those of VSVΔG*LLOV-GP or VSVΔG*EBOV-GP, showing only approximately 10-fold increases.

Human C-type lectins expressed on cell surfaces, such as dendritic cell-specific ICAM-3-grabbing nonintegrin (DC-SIGN) and human macrophage galactose-type C-type lectin (hMGL), are also known to function as attachment factors that promote filovirus infection through interactions with N- and O-linked sugar chains on the GP molecule (51–53). To confirm the involvement of DC-SIGN and hMGL in BatFiloV entry into host cells and to compare their efficiency among filoviruses, we investigated the infectivity of the pseudotyped viruses in cells expressing these C-type lectins. As expected, all tested viruses infected DC-SIGN-expressing cells more efficiently than control cells, although the degree of enhancement varied among the viruses (Fig. 5B). There was no significant difference in infectivity between VSVΔG*EBOV-GP and VSVΔG*LLOV-GP (both showing more than 30-fold increases). Notably, VSVΔG*LLOV-GP exhibited significantly higher efficiency to infect DC-SIGN-expressing cells than three other BatFiloVs (i.e., VSVΔG*BOMV-GP, VSVΔG*MLAV-GP, and VSVΔG*DEHV-GP), which showed approximately 11-fold, 4-fold, and 4-fold higher infectivity in DC-SIGN-expressing cells, respectively, than in control cells. VSVΔG*MLAV-GP and VSVΔG*DEHV-GP exhibited lower efficiency in infecting DC-SIGN-expressing cells than VSVΔG*MARV-GP and VSVΔG*BOMV-GP.

Similar to the findings in DC-SIGN-expressing cells, all the tested viruses showed higher infectivity in hMGL-expressing cells than in control cells (Fig. 5C). As expected, VSVΔG*EBOV-GP, VSVΔG*RESTV-GP, and VSVΔG*MARV-GP showed remarkable increases in infectivity in hMGL-expressing cells (approximately 9-fold, 6-fold, and 12-fold, respectively). By contrast, the infectivity enhancement of VSVΔG*LLOV-GP, VSVΔG*BOMV-GP, VSVΔG*MLAV-GP, and VSVΔG*DEHV-GP in hMGL-expressing cells was significantly lower (4-fold, 2-fold, 3-fold, and 4-fold, respectively) than for VSVΔG*EBOV-GP, VSVΔG*RESTV-GP, and VSVΔG*MARV-GP.

### Cellular tropism of VSIVs pseudotyped with BatFiloV GPs

**Fig 6:**
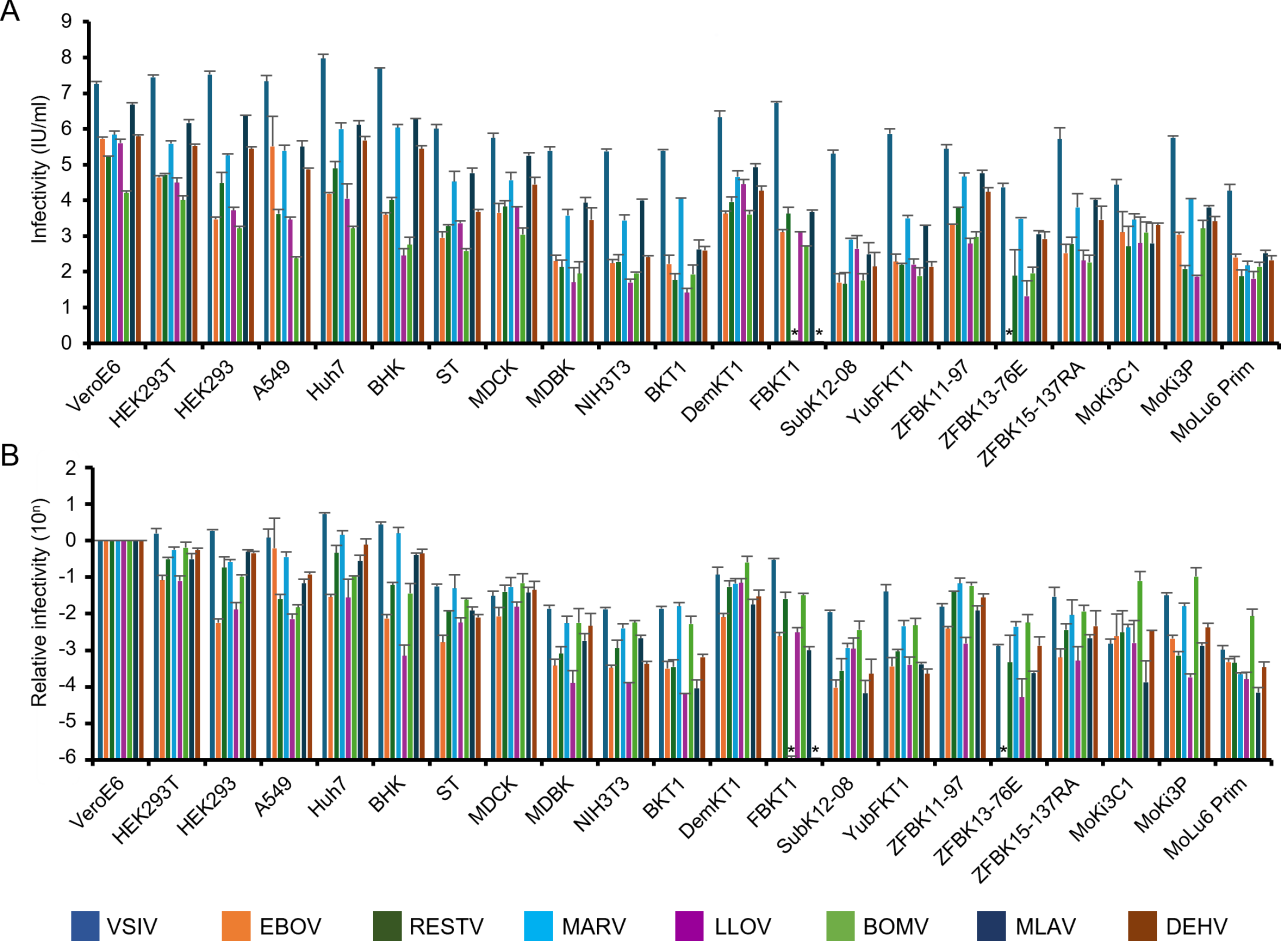
Infectivities of pseudotyped viruses in various mammalian cell lines. VSVΔG*VSIV-G, VSVΔG*EBOV-GP, VSVΔG*RESTV-GP, VSVΔG*MARV-GP, VSVΔG*LLOV-GP, VSVΔG*BOMV-GP, VSVΔG*MLAV-GP, and VSVΔG*DEHV-GP were inoculated into the indicated cell lines, and their IUs in each cell line were determined (**A**). Relative infectivities calculated by setting each IU value in VeroE6 cells to 1 (10^0^) are also shown (**B**). Averages and standard deviations from three independent experiments are shown. Asterisks on FBKT1 and ZFBK13-76E show that virus infectivity was under the limit of detection (20 IU/mL).

To investigate GP-mediated cellular tropism, 21 cell lines of various animal origins, including human and bat cell lines, were infected with the pseudotyped viruses, and infectious units (IUs) were determined for each cell line (Fig. 6A; Table 1). As expected, VSVΔG*VSIV-G uniformly infected these cells at high titers (approximately 10^5^-10^8^ IUs/mL). The viruses pseudotyped with BatFiloV GPs, as well as those with EBOV, RESTV, and MARV, also displayed broad cellular tropism, infecting human, monkey, hamster, pig, dog, bovine, mouse, and bat cell lines. The overall infectivity patterns of VSVΔG*LLOV-GP and VSVΔG*BOMV-GP in these cell lines were similar to those of VSVΔG*RESTV GP and VSVΔG*EBOV-GP, except in the cell line derived from a straw-colored fruit bat (ZFBK13-76E), which is known to be less susceptible to EBOV (54, 55). In contrast, the infectivity patterns of VSVΔG*MLAV-GP and VSVΔG*DEHV-GP resembled those of VSVΔG*MARV-GP. Interestingly, VSVΔG*MARV-GP and VSVΔG*DEHV-GP failed to infect the cell line derived from a Yaeyama flying fox (FBKT1), which is known to have reduced susceptibility to MARV (37, 55), whereas VSVΔG*MLAV-GP successfully infected this cell line (Fig. 6A). When the relative infectivities compared to Vero E6 cells were determined for all cell lines (Fig. 6B), it was also found that VSVΔG*BOMV-GP exhibited increased ability to infect cell lines (MoKi3 C1, MoKi3-P, and MoLu6 Prim) derived from Angolan free-tailed bats, the only known host animal species for BOMV, with 100-fold to 1,000-fold higher titers than the other viruses tested.

**TABLE 1:** Origins of cell lines used in this study

Cell line	Animal	Zoological name	Organ
Vero E6	African green monkey	*Chlorocebus* sp.	Kidney
HEK293T	Human	*Homo sapiens*	Kidney
HEK293	Human	*Homo sapiens*	Kidney
A549	Human	*Homo sapiens*	Lung
Huh-7	Human	*Homo sapiens*	Liver
BHK	Hamster	*Mesocricetus auratus*	Kidney
ST	Pig	*Sus scrofa domesticus*	Testis
MDCK	Dog	*Canis lupus familiaris*	Kidney
MDBK	Cow	*Bos taurus*	Kidney
NIH-3T3	Mouse	*Mus musculus*	Embryo
BKT1	Greater horseshoe bat	*Rhinolophus ferrumequinum*	Kidney
DemKT1	Leschenault’s rousette bat	*Rousettus leschenaultii*	Kidney
FBKT1	Yaeyama flying fox	*Pteropus dasymallus yayeyamae*	Kidney
SubK12-08	The long-fingered bat	*Miniopterus schreibersii*	Kidney
YubFKT1	Eastern bent-winged bat	*Miniopterus fuliginosus*	Kidney
ZFBK11-97	Peter’s epauletted fruit bat	*Epomophorus crypturus*	Kidney
ZFBK13-76E	Straw-colored fruit bat	*Eidolon helvum*	Kidney
ZFBK15-137RA	Egyptian fruit bat	*Rousettus aegyptiacus*	Kidney
MoKi3 C1	Angolan free-tailed bat	*Mops condylurus*	Kidney
MoKi3-P	Angolan free-tailed bat	*Mops condylurus*	Kidney
MoLu6 Prim	Angolan free-tailed bat	*Mops condylurus*	Lung

### Amino acid differences found in GPs among filoviruses and NPC1 loops among cell lines

**Fig 7:**
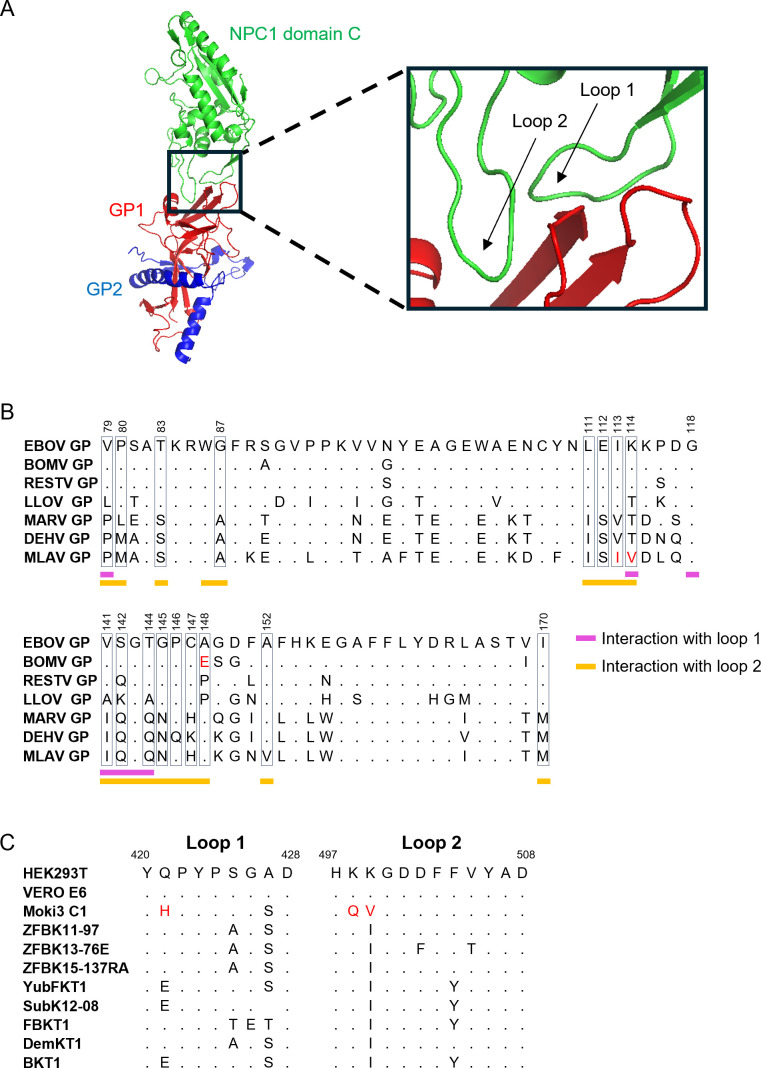
Structure of the GP-NPC1 complex and amino acid sequences of RBD and NPC1-C loops. (**A**) The three-dimensional structure of the EBOV GP and human NPC1-C (PDB ID: 5F1B) interaction is represented in a cartoon model. (**B**) Amino acid sequences of EBOV, BOMV, RESTV, LLOV, MARV, DEHV, and MLAV RBDs are aligned. Amino acid residues that were predicted to interact with the NPC1-C loop 1 and loop 2 regions (61) and varied among the viruses are enclosed by rectangles; positions 79, 80, 83, 87, 111, 112, 113, 114, 141, 142, 144, 145, 146, 147, 148, 152, and 170 (EBOV numbering). The unique amino acid residues observed in MLAV and BOMV GPs are indicated in red. (**C**) Amino acid sequences of NPC1-C loops of HEK293T (NM_000271.5), Vero E6 (XM_037982440.1), MoKi3 C1 (PQ137112.1), ZFBK11-97 (LC462994), ZFBK13-76E (LC462993), ZFBK15-137RA (LC462995), YubFKT1 (LC462271), SubK12-08 (LC462997), FBKT1 (LC462999), DemKT1 (LC462996), and BKT1 (LC462998) are aligned. The unique amino acid residues observed in Moki3 C1 are indicated in red.

It has been shown that the interaction between GP RBD and the NPC1 protein is one of the key determinants of filovirus host tropism (55–57). NPC1, located in late endosomes, acts as the fusion receptor for filovirus entry into cells. This protein contains two loop regions in its domain C (NPC1-C) that interact with GP RBD (Fig. 7A) (29, 30). Thus, amino acid variations at the interface between the NPC1-C loops and GP RBD significantly influence cell susceptibility and host tropism (54–56, 58–60). First, we focused on the molecular mechanism underlying the differential infectivity of VSVΔG*MLAV-GP, VSVΔG*DEHV-GP, and VSVΔG*MARV-GP in FBKT1 cells by comparing amino acid residues in GP RBD. Although the overall similarity among MARV, DEHV, and MLAV was high, we identified two unique amino acid residues in one of the NPC1-C loop-interacting regions of MLAV GP (isoleucine and valine at positions 113 and 114 [EBOV numbering], respectively), which differed from those in MARV and DEHV (Fig. 7B). Isoleucine at position 113 was also found in EBOV, RESTV, BOMV, and LLOV GPs, whereas valine at position 114 was unique to MLAV GP. Next, to understand the molecular basis of the preference of VSVΔG*BOMV-GP for MoKi3 C1, MoKi3-P, and MoLu6 Prim cells, the amino acid sequences of the NPC1-C loops among the human-, monkey-, and bat-derived cell lines used in this study, as well as GP sequences, were compared (Fig. 7C). We found one unique amino acid residue on BOMV GP (glutamic acid at position 148 [EBOV numbering]) and three unique amino acid residues (histidine in loop 1, and glutamine/valine in loop 2) in NPC1 of *Mops condylurus* bat cells.

## DISCUSSION

BatFiloVs are emerging viruses that remain poorly characterized despite their potential to cause disease in humans (40). Studies on their envelope GPs, which play a crucial role in the viral lifecycle, are important for understanding their pathogenicity, ecology, and host range, as well as for clarifying the potential risk as zoonotic pathogens for humans. This knowledge will guide public health interventions to prevent virus spillover and support the development of surveillance strategies and specific medical countermeasures such as vaccines and therapeutics.

Consistent with the phylogenetic relationship among filoviruses based on NP, VP35, and L amino acid sequences (18), GP sequences of BatFiloVs were also divided into two phylogroups, EBOV-like and MARV-like, which include BOMV/LLOV and MLAV/DEHV, respectively. Amino acid sequence comparisons among GPs revealed that BatFiloV GPs generally shared common features with other known mammalian filoviruses, including N-terminal signal peptides, MLDs, furin cleavage sites, conserved cysteine residues, and C-terminal transmembrane/cytoplasmic regions. However, interestingly, two furin cleavage site motifs, RSKR and KKKR, were found in MLAV GP at positions 337–340 and 403–406, respectively. Considering the overall similarity among MLAV, DEHV, and MARV GPs and their multiple alignment data, KKKR at positions 403–406 was assumed to be the primary furin cleavage site of MLAV GP, although further studies are needed to determine the biological significance of the RSKR motif. The primary structures and glycan compositions of MLDs also varied among BatFiloVs. However, since the MLD regions were tentatively predicted in this study solely based on the predicted O-glycosylation sites, additional detailed structural and molecular analyses will be required for a more accurate comparison in the future.

Assuming that bats are the reservoir for all filoviruses, one limitation in comparing BatFiloVs with previously identified human-pathogenic filoviruses is that the latter may harbor mutations associated with host adaptation, potentially influencing pathogenicity and transmissibility. It is currently not possible to address this issue for orthoebolaviruses, since the origins of human-pathogenic viruses (e.g., EBOV and SUDV) have never been isolated from bats. In contrast, sequences of MARVs are available from both bat and human isolates. We compared the amino acid sequences of GPs from MARVs isolated from humans during different outbreaks (Angola, Uganda, and DRC) with those from bats in Uganda and Sierra Leone and found that amino acid residues considered important for potential pathogenicity (e.g., MLD) and host range (e.g., NPC1 binding site) were generally conserved among all variants (data not shown), suggesting limited evidence for the adaptation of bat viruses to humans. Nonetheless, detailed studies of parallel isolates from both reservoirs and spillover hosts would provide critical insights into the mechanisms of host adaptation and virulence of filoviruses.

We found that VLPs consisting of BatFiloV GP, VP40, and NP were morphologically similar to those of EBOV and MARV (37), suggesting common functions of these viral proteins and cellular machinery required to form filamentous particles, whereas the reason for the slightly smaller diameters of BOMV VLPs still needs to be clarified in future studies. Most importantly, although infectious MLAV has not yet been isolated and its morphology remains unverified, our findings strongly suggest that infectious MLAV particles are also expected to be filamentous. It was also demonstrated that mouse antisera to VLPs of respective mammalian filoviruses exhibited limited cross-reactivity to heterologous GP antigens. These results suggest that each BatFiloV GP is serologically distinct from those of the other known mammalian filoviruses, and antigens such as VLPs and purified GPs can serve as target antigens for serological assays to detect specific antibodies against respective BatFiloVs.

Since no epidemics or diseases caused by BatFiloVs have been reported in humans or domestic animals to date, they may not currently pose a major public health problem. However, it is possible that BatFiloV spillover occurs but goes unrecognized. Moreover, in general, the evolution of viruses through a few mutations can alter their host range and pathogenicity. Thus, it is important to know in advance whether previously established therapeutics for EBOV and MARV are also effective against BatFiloVs. Currently, the use of MAbs has gained importance in the treatment of filovirus infections, particularly following the Food and Drug Administration (FDA) approval of two MAb therapies for Ebola virus disease after a randomized controlled clinical trial in the Democratic Republic of the Congo (41), mAb114 (Ebanga, Ridgeback Biotherapeutics, Miami, FL, USA) and REGN-EB3 (Inmazeb, Regeneron Pharmaceuticals, Tarrytown, NY, USA). In our study, some previously established MAbs against EBOV or MARV exhibited the potential to neutralize BatFiloV GP-pseudotyped viruses. Notably, 6D6, a pan-orthoebolavirus MAb targeting the highly conserved internal fusion loop in GP2 (42), neutralized VSVΔG*BOMV-GP and VSVΔG*LLOV-GP due to their shared epitope in this region. This finding demonstrated the extension of its neutralizing capacity to both BOMV, a newly identified member of the *Orthoebolavirus* genus, and LLOV, a virus belonging to the *Cuevavirus* genus. VSVΔG*BOMV-GP was also neutralized by ADI-15946, another cross-reactive MAb that targets a distinct epitope in the base region of GP, crosslinking the GP1 and GP2 subunits, whereas this antibody lacks neutralizing activity against RESTV, likely due to amino acid sequence divergence within its epitope (44). The other tested anti-EBOV MAbs (mAb114, KZ52, 226/8.1, and 133/3.16) were primarily EBOV-specific and failed to neutralize VSVΔG*BOMV-GP. Further investigation for assessing the neutralizing potential of additional MAbs such as MBP047, MBP087, and MBP43, which recognize conserved epitopes on the GP base, heptad repeat, and the membrane-proximal external regions, respectively, as well as polyclonal sera against orthoebolavirus that neutralize EBOV and BOMV (62), will provide more information on conserved epitopes on GPs. It was also observed that anti-MARV GP MR191, which targets RBD, exhibited slight neutralizing activity against VSVΔG*-DEHV-GP, suggesting that the RBD epitope is partially shared between MARV and DEHV. Interestingly, MR191 has been reported to neutralize human immunodeficiency virus-based pseudoviruses bearing MLAV GP (63). By contrast, neutralization against VSVΔG*-MLAV-GP was not observed in our study using the VSIV-based pseudotype system. This was most likely due to the differences in GP incorporation levels per virus particle, as previously suggested (64).

Filoviruses utilize multiple cellular proteins to infect a variety of cells (23). Among these, hTIM-1 and C-type lectins are known as attachment factors/receptors. In this study, we compared the efficiency of hTIM-1- and C-type lectin-mediated entry among BatFiloVs, EBOV, MARV, and RESTV, using pseudotyped viruses. In hTIM-1-expressing cells, VSVΔG*LLOV-GP infected more efficiently than other BatFiloV GP-pseudotyped viruses, confirming that the efficiency of hTIM-1-mediated viral entry varies depending on viral surface GPs, even under the same pseudotyping conditions (65). In DC-SIGN-expressing cells, VSVΔG*LLOV-GP, as well as VSVΔG*EBOV-GP, infected more efficiently than other viruses. We assume that the number of N-linked sugar chains (EBOV ≒ LLOV >BOMV) is critical for the difference among the EBOV-like phylogroups, whereas the structure of sugar chains (i.e., lower amount of high-mannose-type carbohydrate) may explain the reduced ability of the MARV-like phylogroups to infect DC-SIGN-expressing cells. On the other hand, viruses pseudotyped with BatFiloV GPs exhibited weaker infectivity enhancement than those with EBOV, MARV, and RESTV GPs in hMGL-expressing cells, which may be explained by the extent of O-glycosylation with terminal galactose as suggested previously (52). Taken together, these results suggest that the C-type lectin-mediated entry shows different specificities depending on the number and structure of target glycans. Previous studies have suggested that the ability to utilize the C-type lectins to promote cellular entry correlates with differences in pathogenicity among filoviruses (52, 53, 66). The relatively low efficiency of BatFiloVs to utilize C-type lectins may suggest limited potential to infect C-type lectin-expressing cells such as dendritic cells, macrophages, hepatocytes, and endothelial cells, all of which are known as preferred targets of pathogenic filoviruses (23). Accordingly, low pathogenic potential has been demonstrated in animal models for BOMV and LLOV infections (15, 33).

The host range of BatFiloVs remains unknown, although several studies have shown that BatFiloVs have broad cell tropism *in vitro* (11–13, 17, 18, 37). However, these previous studies used only a limited number of bat cell lines and did not directly compare infectivity among BatFiloVs. Using pseudotyped viruses, we confirmed that all BatFiloVs had similar potential to infect a variety of cell lines, including human-derived cells. Interestingly, differences in susceptibility to filoviruses were observed among the bat-derived cell lines. In FBKT1 cells, VSV*G-DEHV-GP and VSV*G-MARV-GP displayed reduced infectivity compared with the other pseudotyped viruses. In contrast, the virus bearing MLAV GP, which also belongs to the MARV-like phylogroup, successfully infected this cell line, likely due to the presence of isoleucine and valine at positions 113 and 114 of MLAV GP (EBOV numbering). The inability of VSVΔG*MARV and VSVΔG*DEHV GP to infect FBKT1 cells could also be explained by unique amino acid residues (threonine, glutamic acid, and threonine at positions 425, 426, and 427, respectively) in NPC1-C loop 1 of FBKT1, as described previously (55). Notably, all Angolan free-tailed bat (*Mops condylurus*)-derived cell lines tested (MoKi3 C1, MoKi3-P, and MoLu6 Prim) exhibited higher susceptibility to VSVΔG*BOMV-GP than to other viruses. This may be explained by unique amino acid residues found in BOMV GP (glutamic acid in a loop 2-interacting region) and NPC1-C of this bat species (glutamine and valine in loop 2), as polymorphisms of NPC1-C loops are a key determinant of filovirus cell tropism (54, 55, 58–60).

In the present study, we focused solely on the biological characterization of BatFiloV GPs; however, the data obtained are insufficient to draw conclusions about the pathogenic potential of these novel filoviruses in humans. Although one option might be to use a surrogate animal model, such as hamsters infected with recombinant VSIV carrying filovirus GP genes (67), it has not been proven that viral pathogenicity in the surrogate model parallels that of authentic viruses. Thus, it is essential to use nonhuman primates and either natural isolates or infectious filoviruses generated by a reverse genetics approach, particularly for MLAV, for which infectious forms are not yet available. The development of reverse genetics systems for this virus could be an effective approach to circumvent this difficulty. Another limitation of our experiments is the lack of mutagenesis studies on GP and NPC1 to confirm the molecular basis of the increased susceptibility of cells derived from the Angolan free-tailed bat to BOMV. Since our previous mutagenesis studies on EBOV, MARV, and LLOV GPs have clarified the molecular mechanisms underlying their host range restrictions in certain bat cell lines (54, 55), we believe that a similar approach is applicable to BOMV GP and NPC1 of this bat species.

The present study provides valuable insights by comparing the biological properties of BatFiloV GPs with those of human-pathogenic filoviruses. Our findings indicate that BatFiloVs share some key characteristics with EBOV and MARV, while suggesting that they may not be as pathogenic as EBOV and MARV. To date, the pathogenicities and transmission routes of these novel filoviruses remain unknown. However, given their affinity for human cell lines, it is crucial to assess the potential risk of human exposure to these viruses. As no human infections have been reported so far, this assessment is especially important in regions where people live in close contact with bats and other wildlife.

## MATERIALS AND METHODS

### Cells

Expi293F (Gibco, Waltham, MA, USA) cells were grown in suspension using Expi293 Expression Medium (Thermo Fisher Scientific, Waltham, MA, USA) at 37°C in 8% CO_2_ with rotation at 125 revolutions per minute (rpm). Human embryonic kidney 293 (HEK293), HEK293T, and HEK293T expressing hTIM-1 (HEK293T-hTIM-1) (65), African green monkey kidney Vero E6, human hepatocellular carcinoma (Huh-7), human lung adenocarcinoma epithelial (A549), baby hamster kidney (BHK), mouse embryo fibroblast (NIH3T3), and Madin-Darby bovine kidney (MDBK) cells were grown in Dulbecco’s modified Eagle’s medium (DMEM) (Sigma-Aldrich, St Louis, MO, USA) supplemented with 10% fetal bovine serum (FBS) (Sigma-Aldrich, St Louis, MO, USA), 100 units/mL penicillin, and 0.1 mg/mL streptomycin. Madin-Darby canine kidney (MDCK) cells were grown in DMEM with 10% calf serum (Gibco, Waltham, MA, USA), 100 units/mL penicillin, and 0.1 mg/mL streptomycin. Swine testis (ST) cells were grown in Eagle’s minimum essential medium (Sigma-Aldrich, St Louis, MO, USA) supplemented with 10% FBS. Bat cells (BKT1, DemKT1, FBKT1, SuBK12, YubFKT1, ZFBK13-76E, ZFBK11-97, and ZFBK15-137RA), human chronic myelogenous leukemia K562 cells, and K562 clones expressing DC-SIGN (K562-DC-SIGN) or hMGL (K562-hMGL) (52, 53) were grown in Rosewell Park Memorial Institute (RPMI) 1640 medium (Gibco, Waltham, MA, USA) supplemented with 10% FBS, 100 units/mL penicillin, and 0.1 mg/mL streptomycin. Other bat cells derived from *Mops condylurus* (MoKi3 C1, MoKi3-P, and MoLu6 Prim) were grown in DMEM/Ham’s F12 (Sigma-Aldrich, St Louis, MO, USA) supplemented with 15% FBS, L-glutamine (Gibco, Waltham, MA, USA), and Anti-Anti (Gibco, Waltham, MA, USA). All these cells were grown at 37°C in a 5% CO_2_ incubator.

### Construction of plasmids expressing GP, NP, and VP40

The nucleotide sequences of BOMV (ON871047), MLAV (NC_055510.1), and DEHV (OP924273.1) were retrieved from GenBank. The coding regions of GP, NP, and VP40 were synthesized in the pUCFa vector (FASMAC Co., Ltd., Kanagawa, Japan), amplified by KOD One PCR master mix (TOYOBO, Osaka, Japan) using specific primers, and cloned into a mammalian expression vector, pCAGGS, using the In-Fusion HD cloning Kit (TAKARA Bio, CA, USA). Plasmids expressing a soluble form of BOMV, MLAV, and DEHV GPs, with the C-terminal His-tagged truncated transmembrane region, were also amplified by KOD One PCR using specific primers containing hexa-His tag sequences and cloned as described above. A designed trimerization motif sequence (GCN3 motif) (68) was inserted between the His tag and residue 640 of DEHV GP to promote trimeric folding. The sequences of all genes were confirmed by Sanger sequencing. The expression plasmids for MARV, EBOV, SUDV, RESTV, TAFV, BDBV, and LLOV were constructed as previously described (37, 69, 70).

### Production and purification of VLPs

Plasmids (pCAGGS) encoding GP, NP, and VP40 of each filovirus (BatFiloVs, TAFV, and RESTV) were used for the transfection of HEK293T cells using TransIT LT-1 (Mirus Bio LLC, WI, USA) according to the manufacturer’s instructions. Forty-eight hours after transfection, the supernatant was collected, and VLPs were purified by ultracentrifugation at 28,000 rpm (SW32Ti rotor, Beckman Coulter) at 4°C for 2 h with a 25% sucrose cushion. VLP pellets were resuspended in phosphate-buffered saline (PBS).

### Pseudotyped viruses

VSIV containing the green fluorescent protein (GFP) gene instead of the receptor-binding VSV G protein gene (VSVΔG*VSIV-G) (22), complemented with GPs of EBOV, SUDV, BDBV, TAFV, RESTV, MARV, LLOV, BOMV, MLAV, and DEHV (VSVΔG*EBOV-GP, VSVΔG*SUDV-GP, VSVΔG*BDBV-GP, VSVΔG*TAFV-GP, VSVΔG*RESTV-GP, VSVΔG*MARV-GP, VSVΔG*LLOV-GP, VSVΔG*BOMV-GP, VSVΔG*MLAV-GP, and VSVΔG*DEHV-GP, respectively), were generated. Infectious units (IUs) of these pseudotyped viruses were determined as described previously (22).

### Virus titration

The background residual infectivity of parental VSVΔG*VSIV-G was abolished before determining the infectivities of pseudotyped viruses by pretreatment with the anti-VSV G MAb VSV-G(N)1-9 (71). K562, K562-DC-SIGN, K562-hMGL, HEK293T, and HEK293T-hTIM-1 grown on 96-well plates were infected with viruses (50-150 IUs/well determined in K562 and HEK293T, respectively). At 20 h postinoculation, GFP-positive cells were counted using an IN-Cell Analyzer 2500 HS (GE Healthcare, Waukesha, WI, USA). The relative percentages of infectivity in hTIM-1-, DC-SIGN-, and hMGL-expressing cells were calculated by setting IUs in control cells (control K562 or HEK293T) to 100%. To determine the infectivities in adherent cell lines of different animal origins, cell monolayers grown on 96-well plates were infected with a serial dilution of VSVs pseudotyped filovirus GPs. At 20 h postinoculation, GFP-positive cells were counted using an IN-Cell Analyzer 2500 HS, and IUs were determined for each cell line. The relative infectivities were calculated by setting IUs in Vero E6 cells to 1.

### Neutralization test

Pseudotyped viruses pretreated with VSV-G(N)1-9 were diluted with DMEM containing 2% FBS to obtain 500–1,500 IUs/50 µL and mixed with an equal volume of serial dilutions of MAbs (6D6, ADI15946, mAb114, KZ52, 133/3.16, 226/8.1, MR78, MR191), followed by incubation for 30 min at room temperature. MAbs 6D6, 133/3.16, and 226/8.1 were obtained from our repository, and ADI15946 (Creative Diagnostics, Shirley, NY, USA), mAb114 (Ridgeback Biotherapeutics, Miami, FL, USA), KZ52 (Absolute Antibody Ltd., Cleveland, UK), and MR78 (ProteoGenix Inc., Morrisville, NC, USA), MR191 (ProteoGenix Inc., Morrisville, NC, USA) were purchased. The mixture (100 µL) was added to a confluent monolayer of Vero E6 cells grown in 96-well plates. Twenty hours later, GFP-positive cells were counted as described above. Percentages of infectivity were calculated by setting IUs in cells infected with each virus alone to 100%.

### Mouse antisera and enzyme-linked immunosorbent assay (ELISA)

Five-week-old BALB/c mice were immunized twice intraperitoneally with purified VLPs (BatFiloVs, TAFV, and RESTV) (100 µg per head) at 3-week intervals. Antisera were collected 14 days after the second immunization. Previously produced antisera to EBOV, SUDV, BDBV, and MARV stored at −80°C were also used (37). Filovirus GP-based ELISA was performed as described previously (69). Soluble forms of EBOV, SUDV, RESTV, TAFV, BDBV, MARV, LLOV, BOMV, MLAV, and DEHV GPs employed as antigens were purified from the supernatant of Expi293F cells transfected with His-tagged GP-expressing plasmids using the Ni-NTA purification system (Invitrogen, CA, USA). Each antiserum was serially diluted with PBS containing 0.05% Tween 20 and 1% skim milk. Bound antibodies were visualized with horseradish peroxidase-conjugated goat anti-mouse IgG (H + L) (Jackson ImmunoResearch Laboratories Inc., USA) and 3,3’,5,5’-tetramethylbenzidine (Sigma-Aldrich, St Louis, MO, USA). The reaction was stopped by adding 1 N phosphoric acid to the mixture, and the optical density at 450 nm (OD_450_) was measured.

### Electron microscopy

Transmission electron microscopy was performed as previously described (37). Purified VLPs (10 µL) fixed with 0.25% glutaraldehyde overnight were placed on collodion-carbon-coated copper grids (Nisshin EM Co. Ltd., Tokyo, Japan) for 2 minutes at room temperature. Then, the grids were washed three times with 10 µL of PBS droplets, negatively stained with 10 µL of 2% phosphotungstic acid hydrate (pH 5.8) (Thermo Fisher Scientific, USA) for 45 seconds, and dried using filter paper. For immunogold staining, we used MAb LGP14-2 for LLOV, MAb ZGP42/3.7 for BOMV and EBOV (37, 69), mouse antisera against MLAV and DEHV VLPs produced as described above, and a 10 nm gold-conjugated goat anti-mouse IgG (H + L) polyclonal antibody (Cytodiagnostics Inc., Burlington, Canada). The purified VLPs (10 µL) were placed onto collodion-carbon-coated copper grids for 10 minutes, followed by blocking with PBS containing 3% BSA for 10 minutes. The grids were then treated with 10 µL of the MAbs or mouse antisera for 30 minutes, washed five times with 10 µL PBS droplets, and incubated with the gold-conjugated goat anti-mouse IgG antibody for 30 minutes. Next, the grids were washed three times with 10 µL PBS droplets and once with 10 µL ultrapure water before being stained with 10 µL 2% phosphotungstic acid hydrate (pH 5.8) for 45 seconds and dried using filter paper. Samples were examined with a transmission electron microscope (HT-7800, Hitachi High-Tech Corporation, Tokyo, Japan) at 80 kV.

### Sequence analyses

The evolutionary history was inferred using the neighbor-joining method (72). Optimal trees based on GP, NP, and L of 10 mammalian filoviruses were constructed. The percentage of replicate trees in which the associated taxa clustered together in the bootstrap test (10,000 replicates) was shown next to the branches (73). The tree was drawn to scale, with branch lengths in the same units as those of the evolutionary distances used to infer the phylogenetic tree. The evolutionary distances were computed using the Poisson correction method (74) and displayed as the number of amino acid substitutions per site. This analysis involved 10 whole amino acid sequences of GP, NP, and L. All ambiguous positions were removed for each sequence pair (pairwise deletion option). Evolutionary analyses were conducted in MEGA11 (75). The amino acid sequences of BatFiloVs used in phylogenetic analysis were retrieved from GenBank as described above. The sequences of EBOV (Mayinga-76), SUDV (Boniface-76), BDBV (Bundibugyo/2007), TAFV (Cote d'Ivoire-95), RESTV (Reston-89), MARV (Angola/2005), and LLOV (Asturias/2003) (GenBank under accession numbers AF086833.2, FJ968794.1, FJ217161.1, U28006, U23152.1, DQ447660.1, and JF828358, respectively) were also used. The potential glycosylation sites were predicted using NetNGlyc-1.0 (https://services.healthtech.dtu.dk/services/NetNGlyc-1.0/) and NetOGlyc-4.0 (https://services.healthtech.dtu.dk/services/NetOGlyc-4.0/) (DTU Health Tech, Lyngby, Denmark), and amino acid residues showing a score above the threshold (0.5) were selected.

### Molecular modeling

Three-dimensional models of the NPC1-C and EBOV GP complex were prepared based on a previous study (61) (Protein Data Bank [PDB] code 5F1B). The three-dimensional structures shown in the figure of this study were prepared using PyMOL (Schrödinger LLC).

## Data Availability

The data that support the findings of this study are openly available in this article and are available from the corresponding author upon request.

## SUPPLEMENTAL MATERIAL

Fig. S1 to S3 jvi.01018-25-s0001.pdf Sequence comparison among filovirus GPs and immunogold staining of VLPs.

ASM does not own the copyrights to Supplemental Material that may be linked to, or accessed through, an article. The authors have granted ASM a non-exclusive, world-wide license to publish the Supplemental Material files. Please contact the corresponding author directly for reuse.
