# Supplementary material for: Comparison of the biological properties of bat-derived filovirus envelope glycoproteins
Source: J Virol. 2025 Sep 25;99(10):e01018-25. doi: 10.1128/jvi.01018-25 (PMC12548439; doi:10.1128/jvi.01018-25)
Supplement: Fig. S1 to S3 — Sequence comparison among filovirus GPs and immunogold staining of VLPs. [file jvi.01018-25-s0001.pdf]

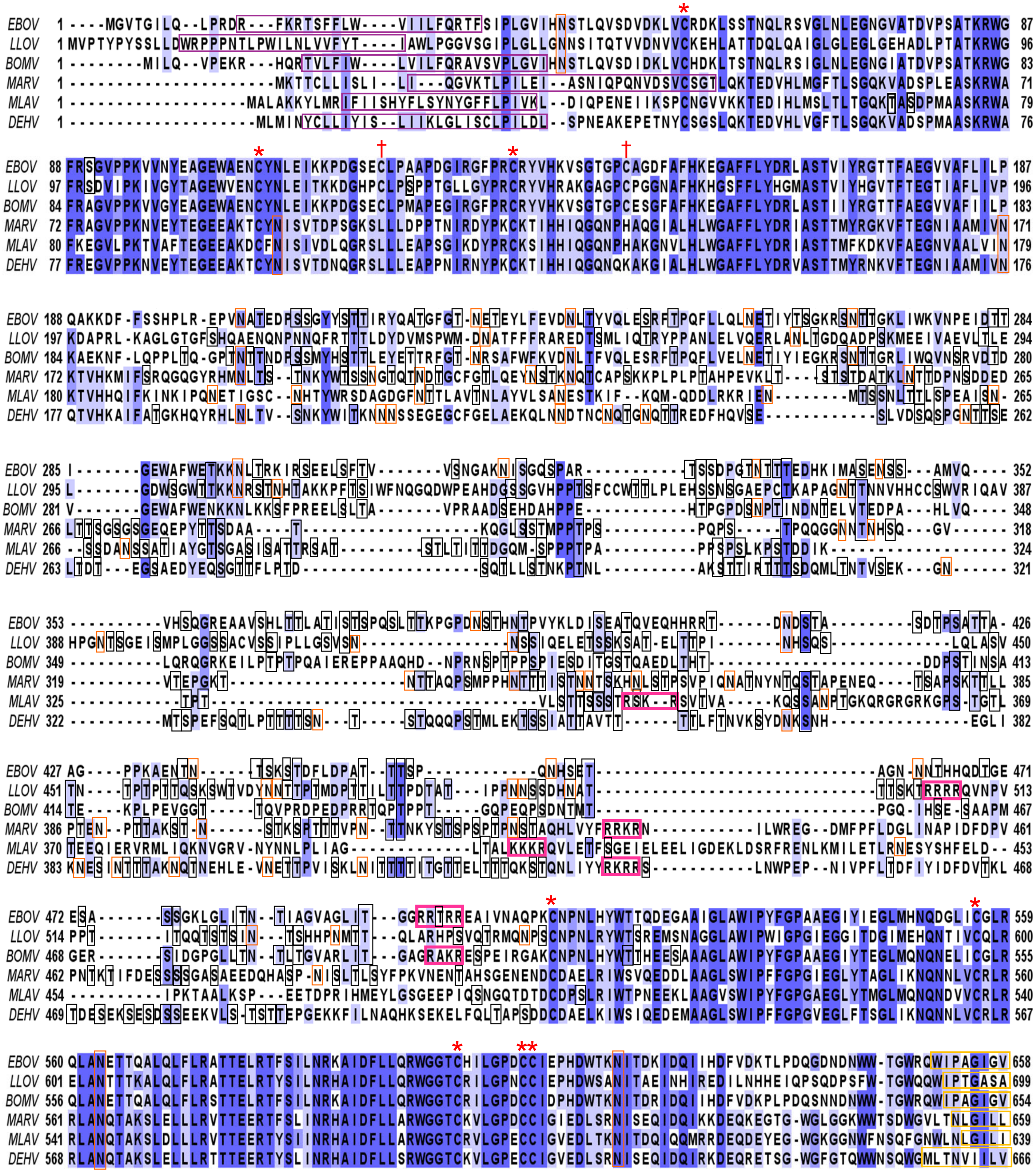

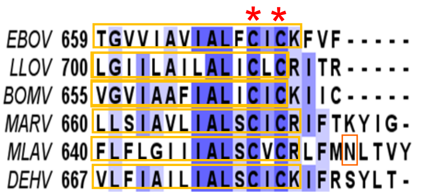

Figure S1. Multiple sequence alignment of BatFiloV, EBOV, and MARV GPs. Complete amino sequences of GP were aligned using the Clustalo Omega multiple sequence alignment tool from EMBL-EB (https://www.ebi.ac.uk/Tools/msa/clustalo/), and the output was edited using Jailview 2.11.2.7. The signal peptide and transmembrane domain framed in purple and yellow were predicted by GENETYX ver.15 and PredictProtein Service version 2.0, respectively. Sequences in pink frames represent furin cleavage motifs. The percent identity is indicated by shades of dark-blue, medium-blue, and light-blue, and none, which indicate 100%, 60%-80%, 40%-60%, and < 40%, respectively. NetNGlyc-1.0 and NetOGlyc-4.0 were used to predict the potential N-glycosylation and O-glycosylation sites, which are framed in orange and black, respectively. Cysteine residues conserved among all tested viruses and those among EBOV, LLOV, and BOMV are indicated by red asterisks and daggers, respectively.

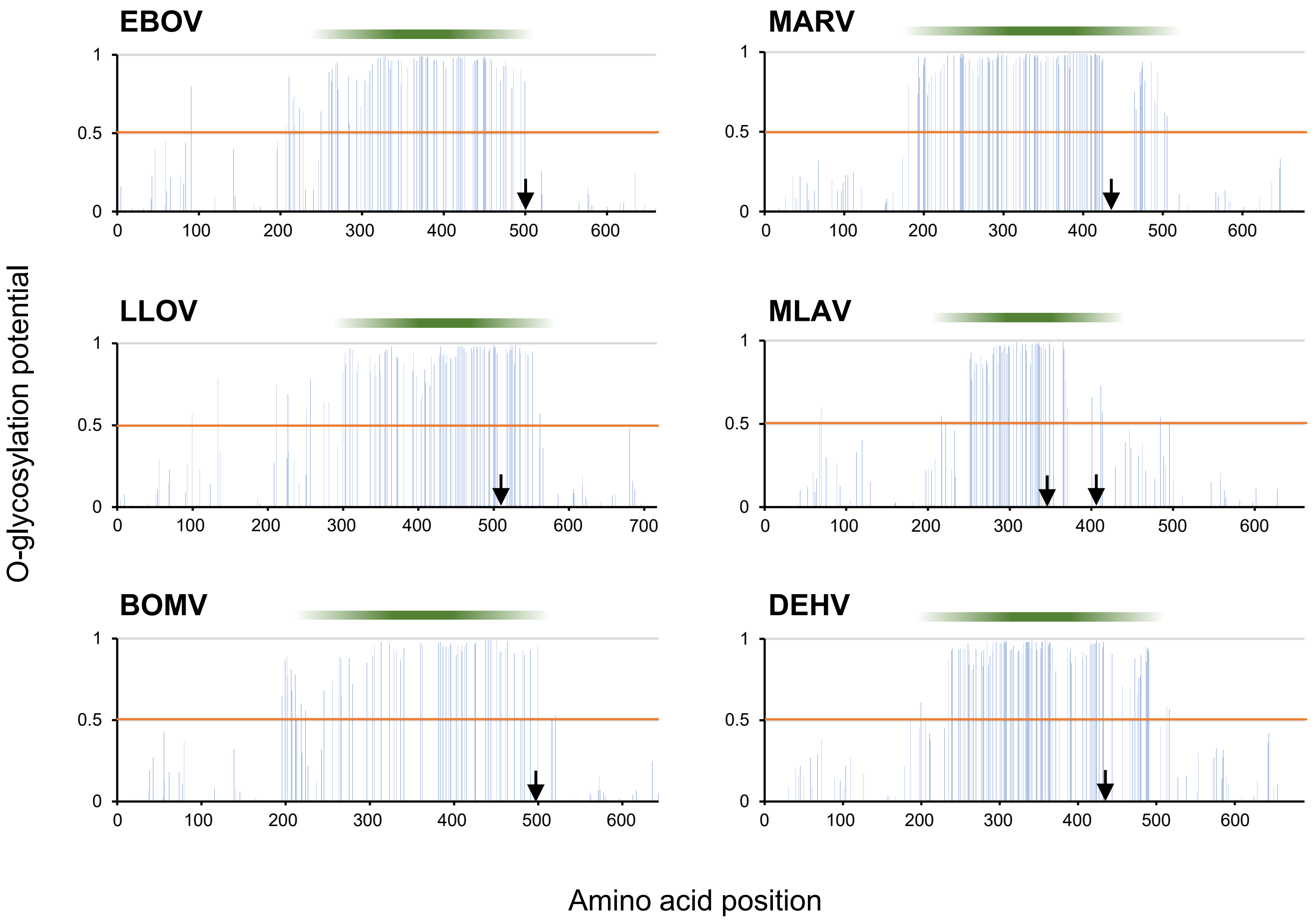

Figure S2. Comparison of EBOV, MARV, and BatFiloV MLDs. Potential O-glycosylation sites were predicted using NetOGlyc-4.0, and approximate regions of MLDs were indicated with green bars. MLDs are previously described for EBOV (35), LLOV (37), and MARV (75). Arrows indicate the cleavage sites.

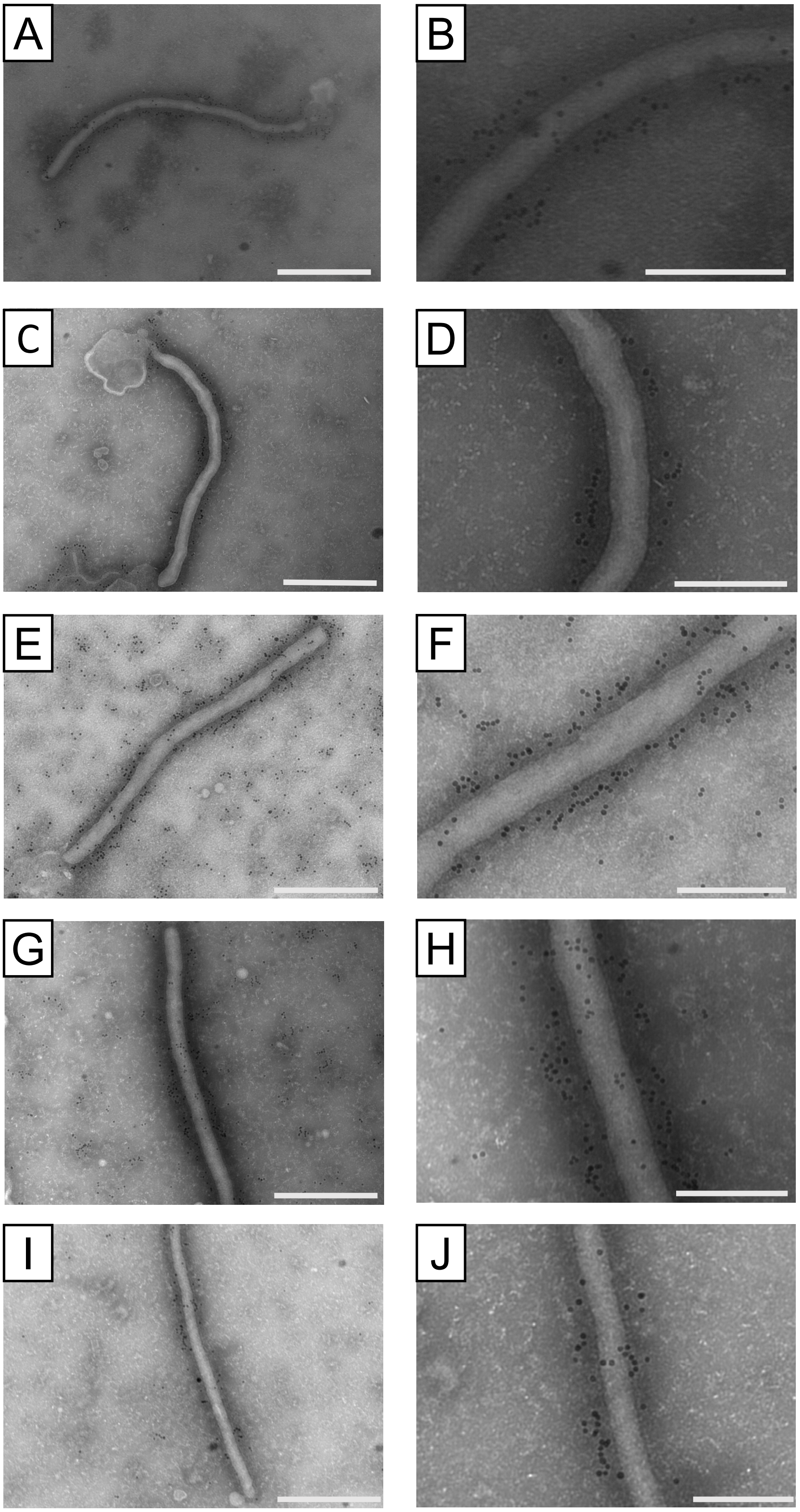

Figure S3. Immunogold staining of VLPs observed by transmission electron microscopy. VLPs purified from the supernatant of HEK293T cells transfected with plasmids encoding GP, NP, and VP40 of LLOV (A, B), BOMV (C, D), MLAV (E, F), DEHV (G, H), and EBOV (I, J) were stained with MAbs or mouse antisera, as described in Materials and Methods. Scale bars: 500 nm (A, C, E, G, I) and 200 nm (B, D, F, H, J).
